# Liang-Ge-San, a Classic Traditional Chinese Medicine Formula, Attenuates Lipopolysaccharide-Induced Acute Lung Injury Through Up-Regulating miR-21

**DOI:** 10.3389/fphar.2019.01332

**Published:** 2019-11-14

**Authors:** Huayi Yang, Zibin Lu, Chuying Huo, Yuyao Chen, Huihui Cao, Pei Xie, Hongling Zhou, Dongyi Liu, Junshan Liu, Linzhong Yu

**Affiliations:** School of Traditional Chinese Medicine, Southern Medical University, Guangzhou, China

**Keywords:** Liang-Ge-San, acute lung injury, miR-21, STAT3, inflammation

## Abstract

**Background:** Acute lung injury (ALI) is a life-threatening disease without effective chemotherapy at present. Liang-Ge-San (LGS) is a famous traditional Chinese medicine formula, which is used to treat ALI in China. However, only a few studies have addressed the mechanisms of LGS in ALI.

**Purpose:** To evaluate the anti-inflammatory effects of LGS on lipopolysaccharide (LPS)-induced ALI, and to explore its underlying molecular mechanism.

**Methods:** Murine RAW264.7 cells were treated with LGS and LPS (1 μg/ml). The generation of IL-6, TNF-α, IL-1β was detected by ELISA. The protein expressions of STAT3 and P-STAT3 (Tyr705) were determined by Western blotting and fluorescence confocal microscopy. STAT3 transcriptional activity was investigated by luciferase reporter gene assay. qPCR was used to detect the expressions of microRNA-21 (miR-21), STAT3, and IL-6. DSS cross-linking assay was used to assess the change of STAT3 dimer. *In vivo* anti-inflammatory effects of LGS were evaluated in an ALI mouse model induced by tracheal instillation of LPS (3 mg/kg). The anti-ALI effects were evaluated by ELISA, qPCR, Western blotting, BCA, and H&E assays.

**Results:** LGS suppressed LPS-stimulated IL-6, TNF-α, and IL-1β generation in murine macrophages RAW264.7. Moreover, LGS down-regulated protein levels of P-STAT3 (Tyr705) and STAT3, inhibited STAT3 transcriptional activity, and up-regulated miR-21. Furthermore, blockage of miR-21 antagonized the inhibitory effects of LGS on the production of IL-6 and the expressions of P-STAT3 (Tyr705) and STAT3 as well as the formation of STAT3 dimer. Critically, LGS up-regulated the expression of miR-21 and inhibited the protein expressions of STAT3 and P-STAT3 (Tyr705) to reduce the release of IL-6 and inflammatory cell infiltration as well as the degree of edema in LPS-induced ALI mice.

**Conclusion:** LGS inhibited LPS-induced ALI through up-regulating miR-21 and subsequently inhibiting the STAT3 signaling pathway, thereby decreasing the release of IL-6.

## Introduction

Acute lung injury (ALI) is a life-threatening syndrome with 30% to 50% mortality, which is mainly caused by sepsis, trauma, and infection. The major characteristic of ALI is excessive and disordered inflammation. The abundant release of pro-inflammatory cytokines and protein-rich edema formation aggravate the pulmonary infectious cascade (Rubenfeld et al., 2005).

The seven members of the STAT signaling pathway (STAT1, STAT2, STAT3, STAT4, STAT5a, STAT5b, and STAT6) are crucial transducers of plentiful cytokines and growth factors, which are predominant for innate immune responses (O’Shea et al., 2015). Among them, STAT3 constitutively activates in inflammation (Villarino et al., 2015). The activation of numerous cytokines and molecules like the anti-inflammatory IL-10, proinflammatory interferon (IFN), interleukin-6 (IL-6), and lipopolysaccharide (LPS), can phosphorylate STAT3 at Tyr705 and induce dimerization and nuclear translocation of STAT3. Subsequently, STAT3 activates the transcription of target genes by binding to the promoters (Johnson et al., 2018). Studies have demonstrated that inhibition of STAT3 pathway significantly suppresses peripheral inﬂammatory responses. Research has proved that STAT3 inhibitors are effective in suppressing inflammation, which is related to diminish infiltration of inflammatory cells, and decrease level of neutrophils, inflammatory cytokines (IL-1β, IL-6, and TNF-α), and chemokines (Zhao et al., 2016). In addition, the suppression of STAT3 signaling pathway can reduce ALI. It has been reported that lung vascular permeability, lung myeloperoxidase accumulation as well as inflammatory cells from serum and bronchoalveolar lavage fluid (BALF) are markedly reduced in the inhibition of STAT3 murine model during ALI (Ju et al., 2015).

It is well accepted that microRNAs (miRNAs) regulate the level of target genes by the translational inhibition and mRNA degradation. Furthermore, increasing studies suggest that miRNAs are closely correlated with ALI. For example, up-regulation of miR-125b improved the survival of ALI mice, and decreased LPS-induced pulmonary inflammation by reducing total cells, neutrophils, and pro-inflammatory cytokines in BALF (Guo et al., 2014). Similarly, upregulation of miR-146a significantly inhibited LPS-induced TNF-α, IL-6, and IL-1β augment in alveolar macrophage NR8383 cells by suppressing levels of IRAK-1 and TRAF-6 in LPS-mediated ALI rat model (Zeng et al., 2013). All these researches reveal that miRNA is a critical regulator for ALI.

Recent studies have been demonstrated that miR-21 can repress inflammation responses. MiR-21 attenuates the levels of inflammatory cytokines, including IL-1β, IL-6, TNF-α as well as infiltration of macrophages *via* suppressing p38 and NF-κB in cardiac inflammation (Yang et al., 2018). Moreover, miR-21 induction in macrophages reduces PDCD4-activated cJun-AP1 which results in elevating expression of anti-inflammatory cytokine IL-10 (Das et al., 2014). However, little is known of the function of miR-21 in ALI.

Liang-Ge-San (LGS) is a famous traditional Chinese medicine formula which was first recorded in “Taiping Huimin Heji Jufang,” a pharmacopeia in Song Dynasty of China. LGS has been empirically used to clear heat and fire for hundreds of years. Currently, it is used to treat ALI, pharyngitis, amygdalitis, and pneumonia in clinic (Wang et al., 2011; Lee et al., 2017). Generally, adult patients take orally 200 ml of LGS decoction at the dosage of 0.195 g/ml twice per day according to the suggestion of traditional Chinese medicine. In previous studies, we have revealed that LGS suppresses ALI by activating the cholinergic anti-inflammatory pathway, which leads to the inhibition of the NF-κB pathway (Liu et al., 2016). Based on our previous research, whether other modulators are associated with anti-inflammatory effects of LGS needs further explored. In this study, we performed in-depth exploration in underlying molecular mechanism related to anti-inflammatory effects of LGS.

## Materials and Methods

### Reagents

The herbs, *Forsythia suspensa* (Thunb.) Vahl (Lot: NO.171102131; origin: Shanxi, China), *Rheum palmatum* L. (Lot: NO.171103591; origin: Sichuan, China), *Scutellaria baicalensis* Georgi (Lot: NO.180106211; origin: Hebei, China), *Gardenia jasminoides* J. Ellis (Lot: NO.171107691; origin: Jiangxi, China), *Glycyrrhiza uralensis* Fisch. ex. DC. (Lot: NO.171201291; origin: Neimenggu, China), *Mentha canadensis* L. (Lot: NO.171207731; origin: Jiangsu, China) were obtained from Kangmei (Guangzhou, China). *Sodium sulfate* (Lot: NO.171207391; origin: Jiangsu, China) were obtained from Kangmei (Guangzhou, China). STAT3 (79D7) Rabbit mAb, HRP-Goat Anti-Rabbit-IgG (H+L)-Conjugate, and HRP-Goat Anti-Mouse-IgG (H+L)-Conjugate were purchased from Cell Signaling Technology (Danvers, USA). Phospho-STAT3 (Tyr705) antibody was obtained from affinity (New York, USA). β-actin antibody was purchased from Boster (Wuhan, China). Alexa Fluor 488-conjugated anti-Rabbit IgG antibody and TRIzol reagent were gained from Invitrogen (Grand Island, USA). 4’, 6-diamidino-2-phenylindole (DAPI) was purchased from Beyotime (Shanghai, China). MiR-21 mimic, miR-21 inhibitor, riboFECT™ CP, miRNA primer of miR-21, as well as U6 were purchased from Ribobio (Guangzhou, China). The sequence of miR-21 inhibitor and miR-21 mimic was as follows: miR-21-5p inhibitor: 5’-UCAACAUCAGUCUGAUAAGCUA-3’; miR-21-5p mimic: 5’-AACAUCAGUCUGAUAAGCUAUU-3’. PrimeScript Tyragent Kit with gDNA Eraser and SYBR^®^ Premix Ex TaqTM II were purchased from Takara (Shiga, Japan). IL-6 and TNF-α ELISA kits were obtained from Dakewei (Beijing, China). BCA protein assay kit and enhanced chemiluminescence (ECL) kit were purchased from Thermo Fisher Scientific (Waltham, USA). Fetal bovine serum (FBS) was purchased from ExCell Bio (Taicang, China). Hematoxylin and eosin were obtained from Yuanye Biotech (Shanghai, China). Paraffin was purchased from Leica (Wetzlar, Germany). Dexamethasone was obtained from Tianxin (Guangzhou, China). Lipopolysaccharide (LPS 055: B5), Thiazolyl Blue Tetrazolium Bromide (MTT), DMEM high glucose medium, disuccinimidyl suberate (DSS), and other reagents were obtained from Sigma-Aldrich (St. Louis, USA).

### Preparation of Chinese Herbal Extracts and Quality Control

All herbs including *Forsythia suspensa* (Thunb.) Vahl, *Rheum palmatum* L., *Scutellaria baicalensis* Georgi., *Gardenia jasminoides* J. Ellis, *Glycyrrhiza uralensis* Fisch. ex. DC., *Mentha canadensis* L., and *Sodium sulfate*. were authenticated by Prof. Ji Ma (Southern Medical University). Voucher specimens (NO. CFV-20190105; NO. CRL-20190106; NO. CSG-20190107; NO. CGE-20190108; NO. CGF-20190109; NO. CMB-20190110; NO. CNS-20190111) were deposited in the Museum of Chinese Traditional Drugs of Nanfan Medical University, Guangzhou, China. All of herbs contained in the formula for Liang-Ge-San were obtained from Kangmei (Guangzhou, China). The quality control and the origin of each herb have been performed in accordance with Chinese Pharmacopoeia (2015 Edition). Besides, there are a quality report of purchased herbs from this GMP supplier (please see **Supplementary Figures** page 4–11). According to the prescription ratio of 4: 2: 1: 1: 2: 1: 2, herbs were weighed and soaked with 10-fold volume of water for 30 min. Subsequently, *Forsythia suspensa* (Thunb) Vahl., *Glycyrrhiza uralensis* Fisch. ex. DC., *Scutellaria baicalensis* Georgi., *Glycyrrhiza uralensis* Fisch. ex. DC., and *Sodium sulfate* were decocted for 10 min. Then, *Rheum palmatum* L. and *Mentha canadensis* L. were added to decoction for another 10 min. Then, the recipe LGS was decocted again with another 6-fold volume of water. Finally, *Sodium sulfate* was added to the aqueous extract. The extract was pooled and further concentrated to 200 ml. Concentrated extract was lyophilized into powder and stored in desiccators. The freeze-dried LGS powder was dissolved in PBS at a concentration of 200 mg/ml and stored at -20°C.

The preparation and HPLC fingerprint analysis of LGS was performed according to previous study (Hu et al., 2014). LGS were separated by a Zorbax Eclipse XDB-C18 column (250 × 4.6 mm, 5 µm, Agilent, United States) and analyzed for specific ingredients and chemical fingerprints as well as quantification of marker compounds of LGS using high-performance liquid chromatography (HPLC, Agilent) and a diode array detector (DAD) (G1315D, VL, Agilent, United States). The mobile phase condition was made up of methyl alcohol in water (A) and 0.1% phosphoric acid in water (B). The column temperature was maintained 38°C, and the flow rate was set to 1 ml/min, with detection wavelength at 235 nm. Eight peaks of LGS extract were identified by comparing the retention times with the reference substances (**Supplementary Figure S1A**). Gardenoside, liquiritin, phillyrin, baicalin, baicalein, rhein, and emodin from China Food and Drug Research Institute were used as reference substances (**Supplementary Figure S1B**). The quantification of marker compounds of Liang-Ge-San was analyzed by HPLC-DAD (**Supplementary Table 1**). The chemical fingerprints and the quantification of marker compounds of LGS had the similar chemical profile to our previous fingerprinting (Hu, 2014; Hu et al., 2014).

### Cell Culture

Murine macrophage cell line RAW 264.7 was purchased from American Type Culture Collection (ATCC, Rockville, USA), and cultured in DMEM supplemented with 10% (v/v) FBS. Cells were cultured in an incubator with 5% CO_2_ at 37°C.

### Cell Viability Assay

Cell viability assay was determined by 3-(4,5-dimethylthiazol-2-yl)-2, 5-diphenyltetrazolium bromide (MTT) assay. RAW 264.7 cells were seeded in a 96-well plate at a density of 8×10^4^ cells/ml and cultured for 24 h. Subsequently, cell viability was analyzed as described previously (Lu et al., 2018).

### Enzyme-Linked Immunosorbent Assay (ELISA)

RAW 264.7 cells were seeded in a 24-well plate at a density of 4×10^5^ cells/ml and cultured for 24 h. Then, Group control (Group CON) was replaced with 500 µl fresh complete medium. Group HLGS (200 µg/ml), Group MLGS (100 µg/ml), Group LLGS (50 µg/ml), and Group DEX (100 µM) were received with different dosage of LGS or DEX in the presence of LPS (1 µg/ml) for additional 24 h respectively. The protein levels of TNF-α, IL-6, and IL-1β in the supernatants or BALF were measured by using the mouse ELISA kits according to the manufacturer’s instructions.

### Western Blot Analysis and Confocal Microscopy

RAW264.7 cells were seeded in 60 mm dishes at a density of 5×10^5^ or confocal dishes at a density of 1×10^5^ for 24 h, respectively. Then, Group CON was replaced with 2.5 ml fresh complete medium. Group of different dosage of LGS were co-cultured with different dosage of LGS (200 µg/ml, 100 µg/ml, 50 µg/ml) and LPS (1 µg/ml) for another 24 h, respectively. The protein expressions of P-STAT3 (Tyr705) and STAT3 were measured by Western blotting. Confocal microscopy was used to confirm the expression levels of STAT3 and P-STAT3 (Tyr705). Cells were co-incubated with LGS (200 µg/ml) and LPS (1 µg/ml) for 24 h. Western blotting and confocal microscopy were performed as described previously (Lu et al., 2018). The protein levels of Western blotting were quantified by ImageJ 1.4.3 (National Institutes of Health, USA).

### Detection of STAT3 Luciferase Activity

After constructing pAdeno-Stat3 promoter-Luciferase and pAdeno-MCMV-Renilla-IRES2-EGFP adenovirus by using plasmid, RAW264.7 cells were seeded in 96-well plates at a density of 5000/well. After 24 h, pAdeno-MCMV-Renilla-IRES2-EGFP adenovirus (virus titer: 2.00×10^10^ PFU/ml; final titer 1.6×10^7^ PFU/ml) and pAdeno-Stat3 promoter-luciferase adenovirus (virus titer: 2.00×10^10^ PFU/ml; final titer 2.00×10^8^ PFU/ml) were transfected into cells. After 24 h incubation, the medium of Group CON was replaced. RAW264.7 cells co-cultured with LGS (200 µg/ml) and LPS (1 µg/ml) for 24 h in Group LGS. Then the luciferase activity was detected according to the Promega protocol (Madison, USA).

### Transient Transfection

RAW264.7 cells were seeded in 60 mm cell culture dishes at a density of 2×10^5^ and cultured for 24 h. After washed three times with PBS, miR-21 mimic (50 nM) or miRNA-21 inhibitor (200 nM) were transfected into cells with riboFECT™ CP Reagent according to manufacturer’s instruction.

### Real-Time Quantitative Polymerase Chain Reaction (qPCR)

Total RNA was extracted from cells (2×10^7^) or tissues (∼30 mg) using TRIzol™ reagent. Total RNA was reversely transcribed into cDNA by using Takara PrimeScript™ RT reagent kit. Subsequently, the sample was implemented according to the instruction of Takara SYBR^®^ Premix Ex TaqTM II (Tli RNaseH Plus).

### Detection of STAT3 Dimer

RAW264.7 cells were seeded in six-well plates at a density of 2×10^5^ for 24 h. After 24 h incubation, miR-21 mimic (50 nM) or miRNA-21 inhibitor (200 nM) were transfected into cells. Then, Group CON was replaced with 2.5 ml fresh complete medium, and the transfected cells were exposed to LGS and LPS (1 µg/ml) for another 24 h. After 24 h incubation, cells (2×10^7^) were washed twice with PBS, and resuspended in PBS. DSS at a final concentration of 0.5 mM was added to reaction for 30 min at RT. Subsequently, the equal volume of Tris-HCl (pH = 7.5) was added at a final concentration of 20 mM for 15 min at RT to terminate the DSS cross-linking reaction. Then, cells were centrifuged at 500 ×g for 5 min at 4°C. The supernatant was discarded and total cellular proteins were extracted. The formation of STAT3 dimer was detected by Western blotting.

### Animals

Male BALB/c mice (18–22 g) were obtained from the Center of Experimental Animals of Southern Medical University (Guangzhou, Guangdong, China). All experiments were executed according to the National Institutes of Health guide for the care and use of laboratory animals was approved by the ethical committee for the experimental use of animals at Southern Medical University (L2018119).

### Grouping and Modeling

Forty-eight mice were randomly divided into six groups (n = 8/group). A co-worker blinded to the experimental protocol randomized animals into groups: control (CON), LPS, high dose of LGS (HLGS), medium dose of LGS (MLGS), low dose of LGS (LLGS), dexamethasone (DEX). Group CON was treated with normal saline (1 ml/day, intragastric). Group HLGS, Group MLGS, Group LLGS, and Group DEX were received with a high dosage of LGS (15 g/kg/day, intragastric), medium dosage of LGS (10 g/kg/day, intragastric), low dosage of LGS (5 g/kg/day, intragastric) or DEX (10mg/kg/day intraperitoneal injection) for constitutive 7 days respectively. After 7 days, all groups except group CON were treated with LPS (3 mg/kg, intratracheal) for 8 h to induce the ALI murine model. The mice were anesthetized with 1.5% (*w*/*v*) pentobarbital sodium solution to acquire BALF samples and lung tissues.

### Histopathological Observation of Lung Tissues

Right lung tissues were fastened with 10% (*w*/*v*) paraformaldehyde for 24 h. Then, the lung tissues were rinsed with water and dehydrated in ascending series of ethanol, cleared in xylene. After paraffin embedding, 5-µm thick sections were stained with hematoxylin and eosin (Mulloy et al., 2012). The samples were observed by light microscopy (Olympus IX 53, Tokyo, Japan) and histopathological scores were assessed. The lung sections were assessed according to the degree of interstitial infiltration, total neutrophil counts per high power field, and extent of alveolar edema. Each section was assigned using the following grading (Reece et al., 2005): A. neutrophils per high-powered field (Score: 0 = < 5 cells, 1 = 6–10 cells, 2 = 11–20 cells, 3 = > 20 cells), B. interstitial infiltration (Score: 0 = none, 1 = minimal, 2 = moderate, 3 = severe), C. alveolar edema (Score 0: = < 5%, 1 = 6%–25%, 2 = 26%–50%, 3 = > 50%). The histological score was calculated by summation of these three grading.

### Lung Wet/Dry Weight (W/D) Ratio

The freshly harvested lung tissues were excised, blotted dry, and weighed using an electronic balance (Sartorius) to obtain the wet weight (W), and then dried in an oven at 60°C to obtain the dry weight (D) until the weight was not changed. To assess the degree of pulmonary edema, W/D ratio was determined by the following formula: W/D × 100%.

### Protein Levels in BALF

After the sacrifice of mice, the protein concentration in BALF was determined by the BCA protein assay kit.

### Statistical Analysis

Data were analyzed by GraphPad Prism software (version 5.0; GraphPad Software Inc) and expressed as mean ± SEM at least three independent experiments. Comparison of means among multiple groups was accomplished by one-way analysis of variance (ANOVA). Tukey’s test was used for multiple comparisons. Student’s *t*-test was used to compare two independent groups. *P* ≤ 0.05 were considered as statistically significant.

## Results

### LGS Inhibits the Release of IL-6, TNF-α, and IL-1β in RAW264.7 Macrophage Cells

**Figure 1:**
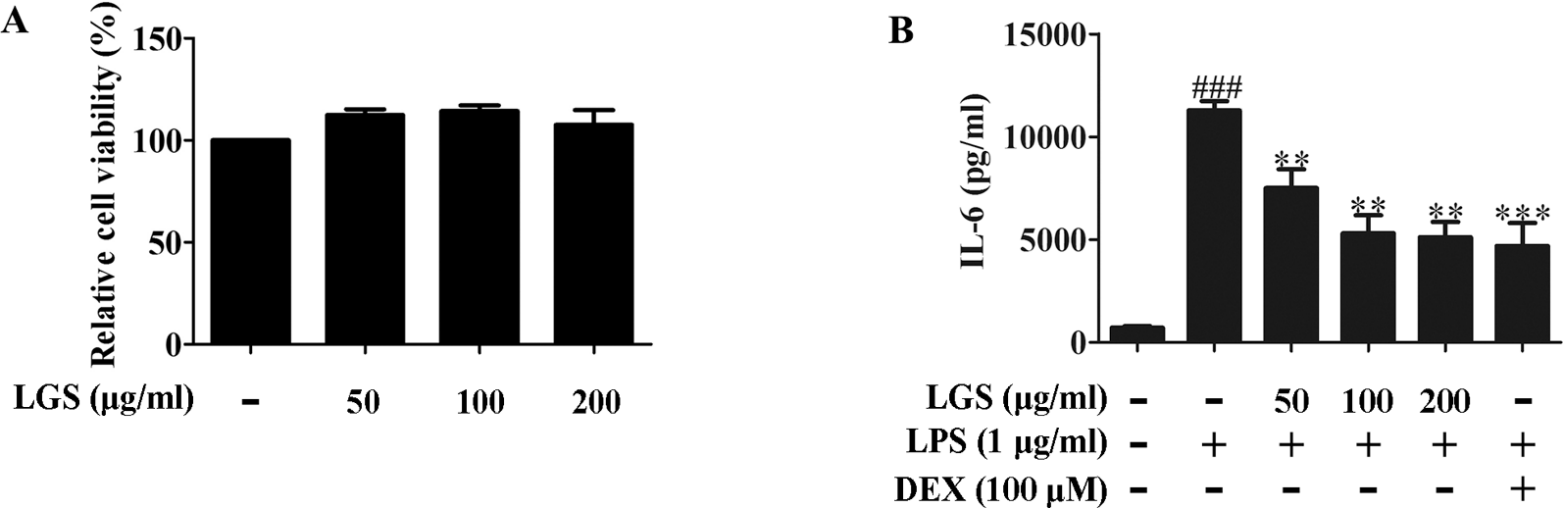
LGS suppresses the release of IL-6 in LPS-induced RAW264.7 cells. **(A)** LGS (50−200 μg/ml) has no obvious cytotoxicity in RAW264.7 macrophage cells. The cell cytotoxicity of LGS was confirmed by MTT assay. **(B)** LGS inhibits the production of IL-6 in LPS-induced RAW264.7 cells. Macrophages were cultivated with LGS (200 μg/ml) or dexamethasone (DEX, as a positive control) (100 μM) in the presence of LPS (1 μg/ml) for 24 h. The level of IL-6 in supernatants was analyzed by ELISA. Data are represented as the mean ± SEM of three independent experiments. ^###^ *P* < 0.001 *versus* control, **P < 0.01, ***P < 0.001 *versus* LPS treatment by one-way ANOVA with Tukey’s test.

We first investigated the cytotoxicity of LGS in RAW264.7 cells. Briefly, cells were treated with various concentrations of LGS for 24 h and the cell viability was measured by MTT assay. As showed in **Figure 1A**, LGS (50−200 µg/ml) had no obvious cytotoxicity in RAW264.7 cells. Therefore, LGS at these concentrations was chosen in the subsequent experiments.

It’s well accepted that LPS can stimulate the generation of pro-inﬂammatory cytokines IL-6 and TNF-α in RAW264.7 macrophage cells, which contribute to inﬂammation (Cheng et al., 2014). To verify the anti-inflammatory effects of LGS, we examined the concentrations of IL-6, TNF-α, and IL-1β in the supernatants of RAW264.7 cells after LGS and LPS treatments. The results showed that exposure of RAW264.7 cells to LPS (1 µg/ml) increased the productions of IL-6, TNF-α, and IL-1β. However, these pro-inflammatory cytokines were significantly inhibited after LGS treatment. Moreover, LGS at the concentration of 200 µg/ml showed the similar anti-inflammatory activity compared with that of dexamethasone (DEX) (100 µM) (**Figures 1B** and **S2**), which was consistent with our previous research (Liu et al., 2016). We also investigated the cytotoxicity and the anti-inflammatory activity of marker compounds of LGS in RAW264.7 cells. Results showed that these compounds at a non-toxic concentration can inhibit the release of IL-6 in LPS-stimulated RAW264.7 cells (**Supplementary Figure S3**). Taken together, these findings indicated that LGS exerts anti-inﬂammatory effects in LPS-induced RAW264.7 macrophage cells.

### LGS Attenuates STAT3 Activation in LPS-Stimulated RAW264.7 Cells

**Figure 2:**
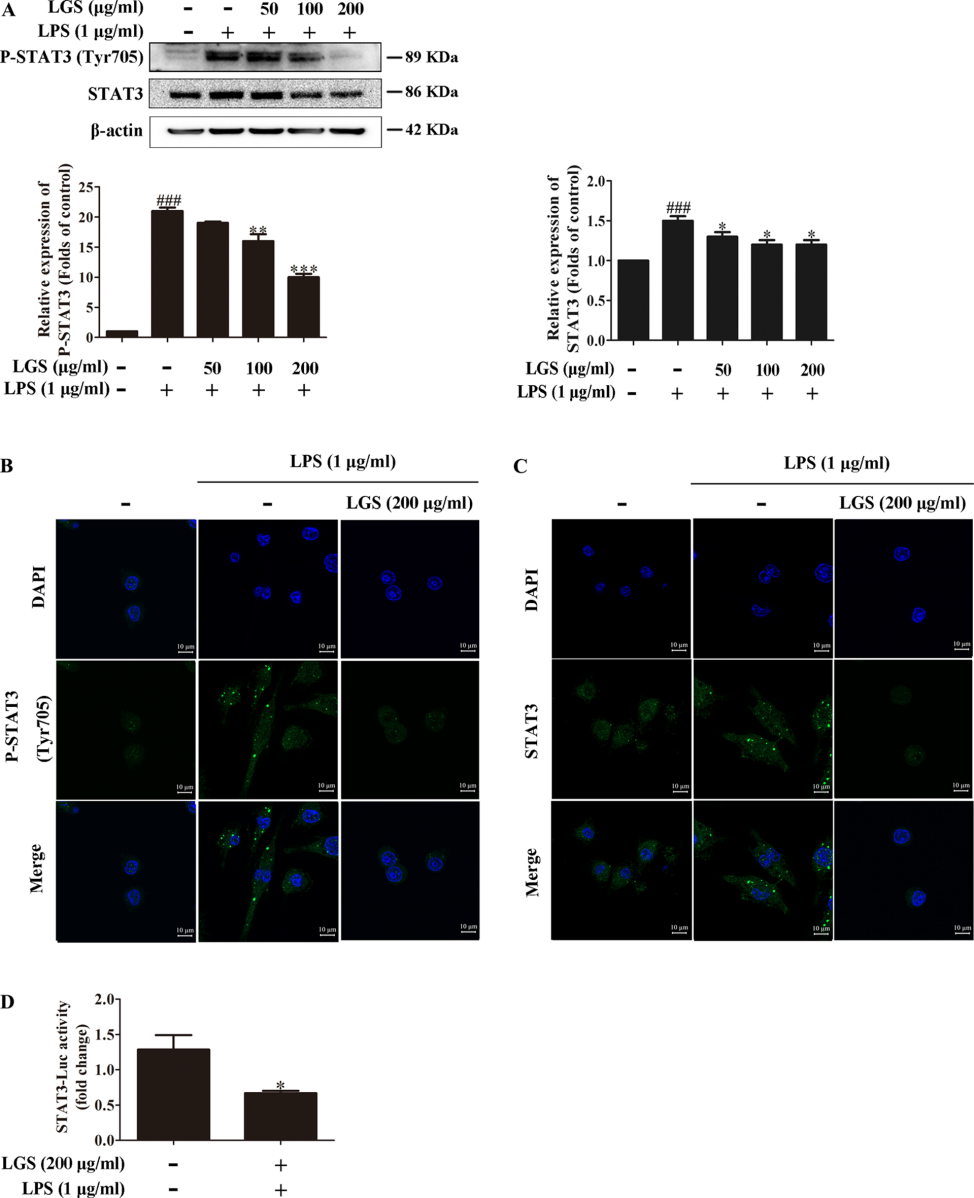
LGS inhibits the STAT3 activation in LPS-induced RAW264.7 cells. **(A−C)** LGS inhibits the STAT3 signaling pathway. RAW264.7 cells were cultured with various concentrations of LGS in the presence or absence of LPS (1 μg/ml). The protein expressions of P-STAT3 (Tyr705) and STAT3 were measured by Western blotting **(A)** and confocal microscopy (600 ×) **(B** and **C)**, respectively. ^###^ *P* < 0.001 *versus* control, *P < 0.05, **P < 0.01, ***P < 0.001 *versus* LPS treatment by one-way ANOVA with Tukey’s test. **(D)** LGS suppresses the transcriptional activity of STAT3. The RAW264.7 cells were incubated in 96-well plates for 24 h. After infected by adenovirus, cells were treated with LGS (200 μg/ml) for another 24 h. The luciferase activity was detected by Dual-Luciferase^®^ Reporter Assay System. Data are represented as the mean ± SEM of three independent experiments. *P < 0.05 *versus* control by Student’s *t*-test.

Considered that the release of IL-6 was more obvious than that of TNF-α, we further explored the influence of LGS on STAT3 pathway, which is a critical regulator of IL-6. Western blotting showed that LPS stimulation induced the phosphorylation of STAT3 at Tyr705 and increased the protein expression of STAT3 in RAW264.7 cells, whereas LGS treatment prevented these phenomena (**Figure 2A**). In addition, In **Figure 2B**, p-STAT3 was increased and distributed evenly in the cytoplasm and nucleus in LPS-induced RAW 264.7 cells. After LGS treatment, p-STAT3 was decreased and mainly distributed in the cytoplasm. In **Figure 2C**, the expression level of STAT3 was significantly decreased in both nuclear and cytosol after LGS treatment compared with LPS group. These data further confirmed the result of **Figure 2A**. Luciferase reporter assay result revealed that LGS suppressed transcriptional activity of STAT3 in LPS-stimulated RAW264.7 cells (**Figure 2D**). Taken together, these findings demonstrated that LGS attenuates inﬂammation by inhibiting the STAT3 signaling pathway.

### LGS Suppresses the STAT3 Pathway *Via* Up-Regulating MiR-21

**Figure 3:**
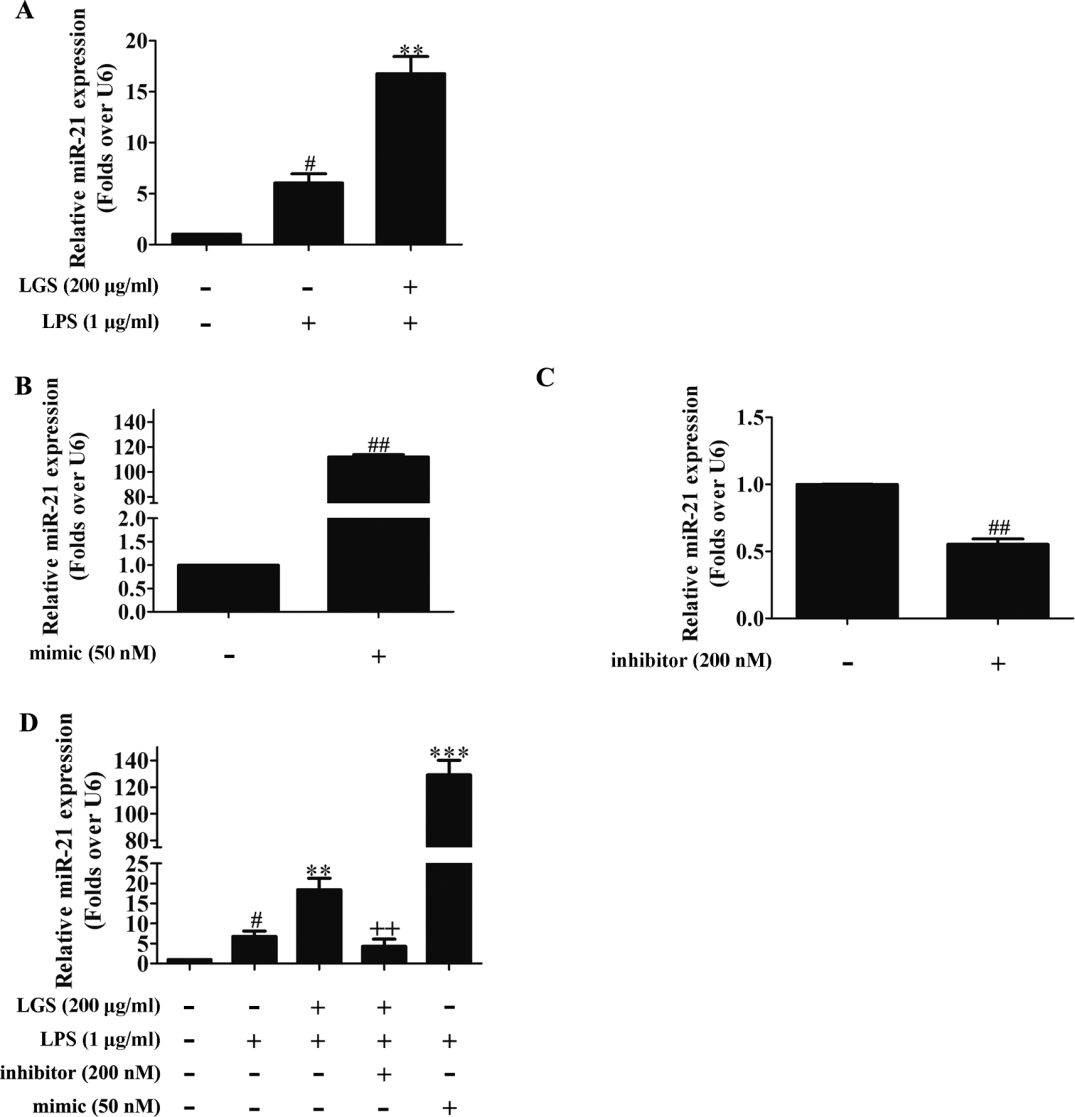
LGS up-regulates miR-21 in RAW264.7 cells. **(A)** LGS enhances the expression of miR-21. Cells were incubated with LGS (200 μg/ml) and stimulated with LPS (1 μg/ml) for 24 h. **(B−C)** The function verification of miR-21 mimic and inhibitor. Cells were transfected with miR-21 mimic (50 nM) or inhibitor (200 nM) for 24 h. ^##^ *P* < 0.01 *versus* control by Student’s *t*-test. **(D)** miR-21 inhibitor counteracts the up-regulation of miR-21 after LGS treatment in LPS-stimulated RAW264.7 cells. After cells were transfected with miR-21 mimic or inhibitor for 24 h, LGS (200 μg/ml) and LPS (1 μg/ml) were added to the RAW264.7 cells for 24 h. The expression of miR-21 was measured by qPCR (A−D). Data are represented as the mean ± SEM of three independent experiments. ^#^ *P* < 0.05 *versus* control, **P < 0.01, ***P 0.001 *versus* LPS treatment, ^++^ *P* < 0.01 *versus* LGS treatment by one-way ANOVA with Tukey’s test.

MicroRNAs play a pivotal role in anti-inﬂammatory response. Abundant evidences have proved that many microRNAs are involved ALI, such as miR-100, miR-133a, miR-140 (Ferruelo et al., 2018). Therefore, we next performed studies to identify which microRNAs regulate STAT3. Among the microRNAs that are highly associated with STAT3 (TargetScan, Release 7.2), further qPCR data showed that miR-17, miR-21, and miR-124 were increased after LGS treatment. Nevertheless, we found that miR-21 was increased most significantly after LGS treatment (**Figure S4**). Previous studies have confirmed that miR-21 attenuates inflammation through inhibiting p38 and NF-κB in RAW264.7 (Yang et al., 2018). Therefore, we further explored that whether LGS could increase the expression of miR-21 in LPS-stimulated RAW264.7 cells. As showed in **Figure 3A**, LGS up-regulated the expression of miR-21 in RAW264.7 cells by qPCR. We next utilized miR-21 mimic and inhibitor to up-regulate or down-regulate the expression of miR-21, respectively (**Figures 3B, C**). The results showed that the miR-21 inhibitor counteracted the up-regulation of miR-21 after LGS treatment in LPS-stimulated RAW264.7 cells (**Figure 3D**).

**Figure 4:**
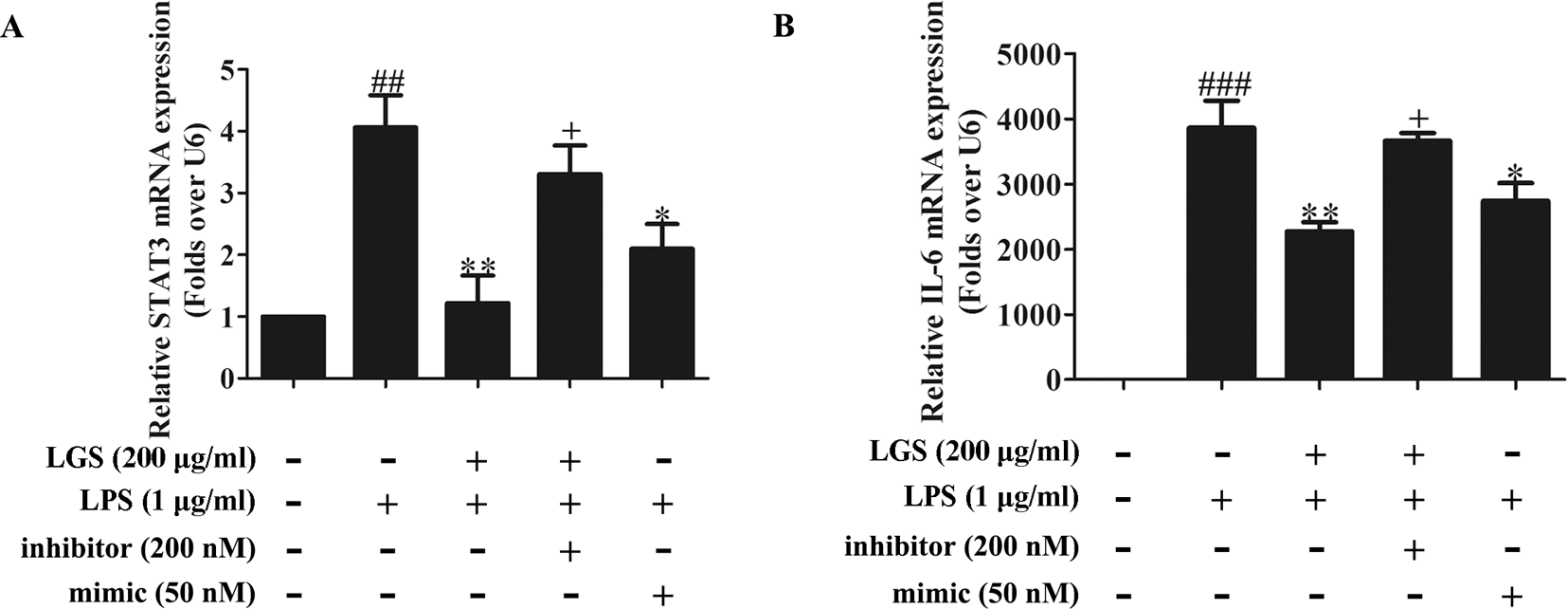
The blockage of miR-21 abolishes the inhibitory effects of LGS on STAT3 mRNA **(A)** and IL-6 mRNA **(B)**. The cells were transfected with miR-21 mimic or inhibitor for 24 h. After transfection, macrophages were cultured with LGS (200 μg/ml) and LPS (1 μg/ml) for another 24 h. The mRNA expressions of STAT3 and IL-6 were measured by qPCR. Data are represented as the mean ± SEM of three independent experiments. ^##^ *P* < 0.01 and ^###^ *P* < 0.001 *versus* control, ^*^ *P* < 0.05, ^**^ *P* < 0.01 *versus* LPS treatment, ^+^ *P* < 0.05 *versus* LGS treatment by one-way ANOVA with Tukey’s test.

**Figure 5:**
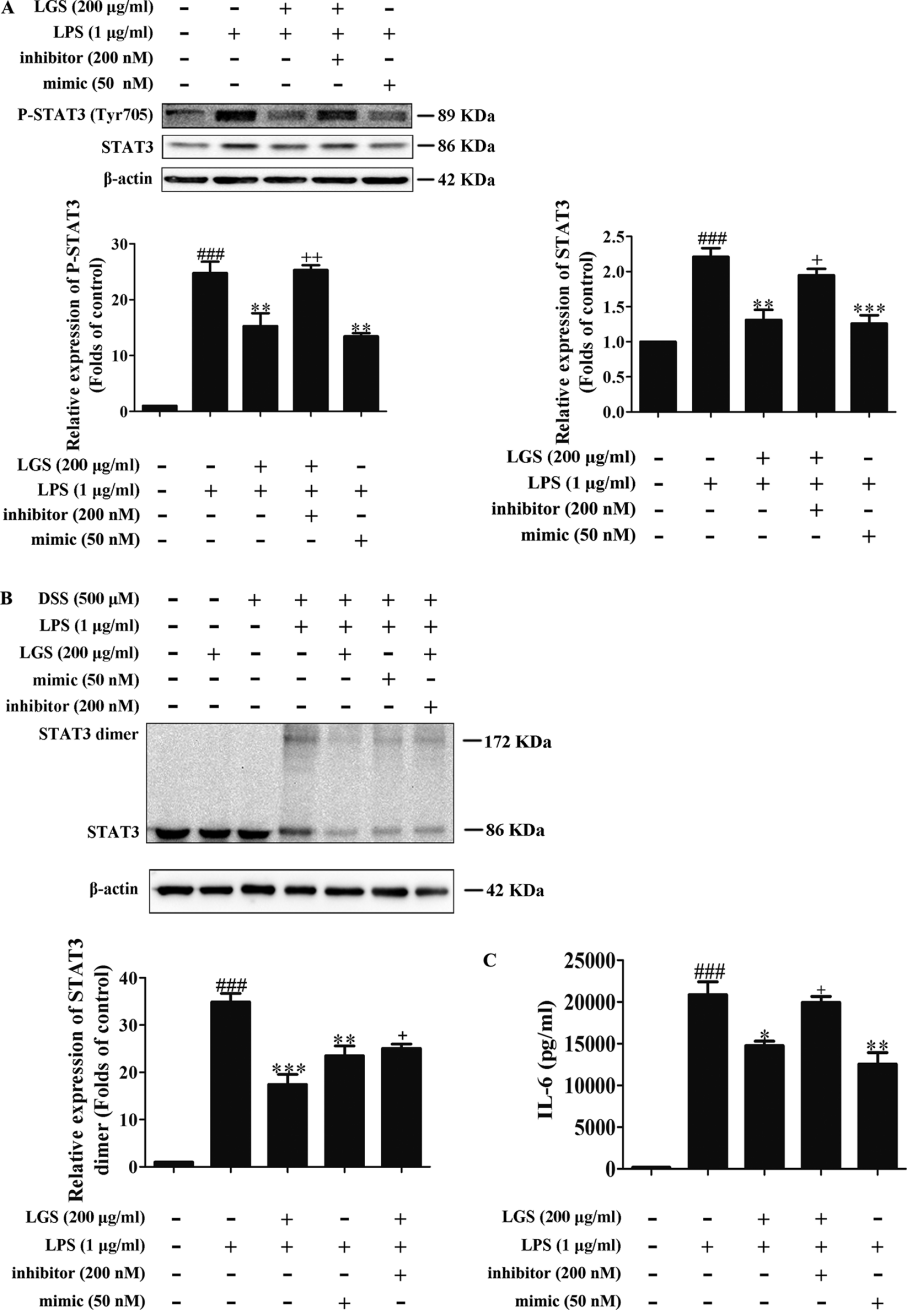
The blockage of miR-21 attenuates inhibitory effects of LGS on the STAT3 signaling pathway. The cells were transfected with miR-21 mimic or inhibitor for 24 h. After transfection, macrophages were incubated with LGS (200 μg/ml) and LPS (1 μg/ml) for another 24 h. **(A)** The inhibition of miR-21 decreases the inhibitory effects of LGS on the protein expressions of P-STAT3 (Tyr705) and STAT3. The protein expressions were measured by Western blotting. **(B)** The inhibition of miR-21 attenuates the inhibitory effect of LGS on STAT3 dimer. DSS was used to cross-link the protein and Western blotting was used to measure the dimer content of STAT3. **(C)** The inhibition of miR-21 increases the level of IL-6. The generation of IL-6 was detected by ELISA. Data are represented as the mean ± SEM of three independent experiments. ^###^ *P* < 0.001 *versus* control, *P < 0.05, **P < 0.01 and ***P < 0.001 *versus* LPS treatment, ^+^ *P* < 0.05, ^++^ *P* < 0.01 *versus* LGS treatment by one-way ANOVA with Tukey’s test.

To further elucidate the role of miR-21 in anti-inﬂammatory effects of LGS, we suppressed miR-21 in RAW264.7 cells and examined the mRNA expressions of STAT3 and IL-6. As confirmed by qPCR, miR-21 inhibitor attenuated the suppressive effects of LGS on the mRNA levels of STAT3 and IL-6 (**Figures 4A, B**). Moreover, we further found that miR-21 mimic exhibited the similar effect as LGS, whereas the miR-21 inhibitor abolished the inhibitory effects of LGS on P-STAT (Tyr705) and STAT3 (**Figure 5A**). The phosphorylation of STAT3 can result in the dimerization and the nuclear translocation of STAT3 to induce the transcription of IL-6. Therefore, we investigated the protein expression level of STAT3 dimer. As showed in **Figure 5B, L**GS as well as miR-21 mimic, attenuated the protein expression of STAT3 dimer in RAW264.7 cells. Conversely, miR-21 inhibitor reduced this effect of LGS. Consistently, the inhibition of miR-21 could also decrease the suppressive effect of LGS on the level of IL-6 (**Figure 5C**).

Collectively, these results demonstrated that LGS inhibits inﬂammation by the STAT3 signaling pathway through the up-regulation of miR-21 *in vitro*.

### LGS Attenuates Inﬂammation in ALI Mice Through Up-Regulating miR-21

**Figure 6:**
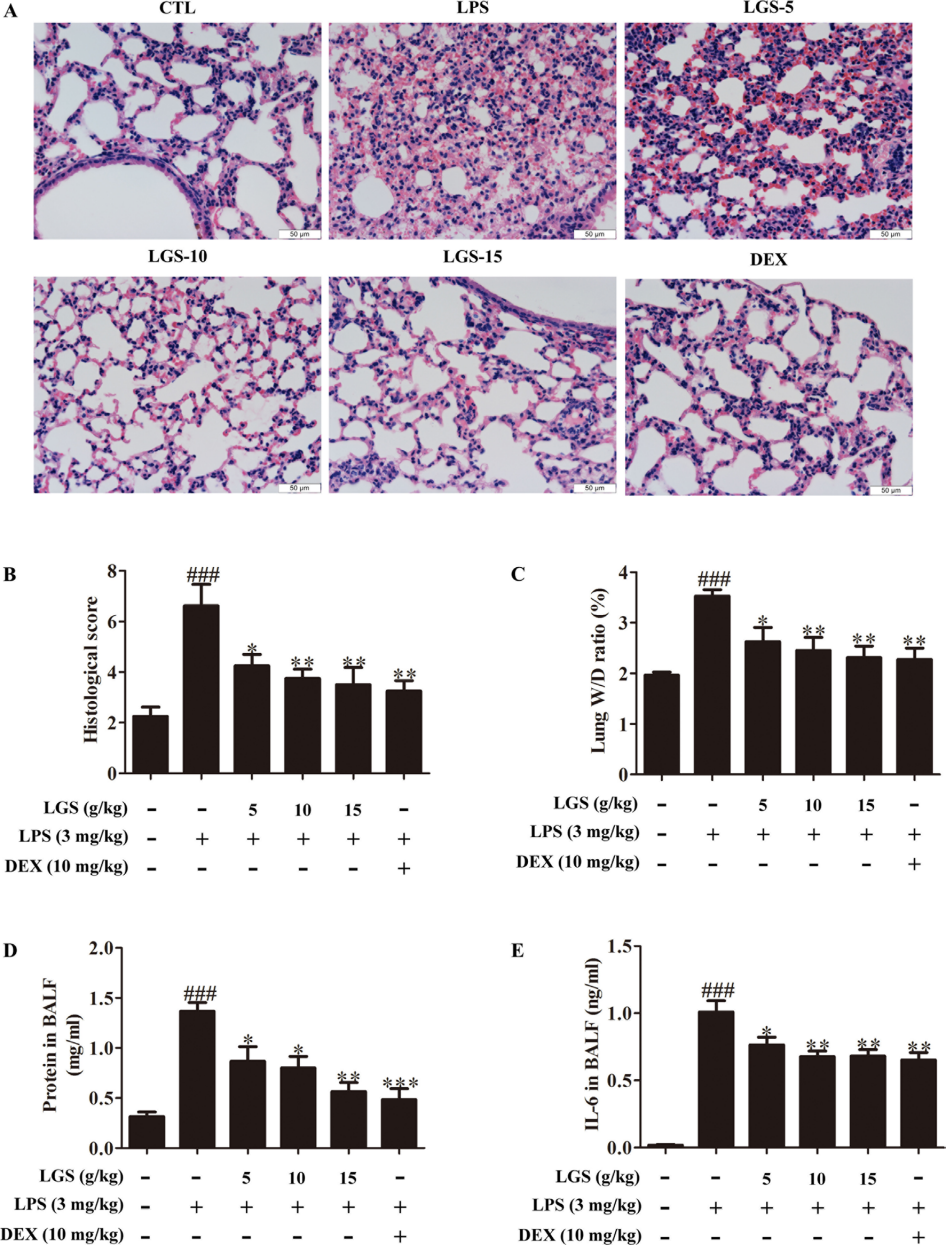
LGS attenuates acute lung injury in mice. After being intragastric administered with LGS (5 g/kg, 10 g/kg, 15 g/kg) or DEX (10 mg/kg) for 7 days, respectively. LPS (3 mg/kg) was intratracheally instilled to induce the ALI model. The mice were anesthetized with 1.5% (*w*/*v*) pentobarbital sodium solution to acquire bronchoalveolar lavage fluid (BALF) samples and lung tissues. **(A−B)** LGS ameliorates the pathological changes in ALI mice. After H & E staining, the tissue sections were observed (**A**, 400 ×) and the histological score were estimated **(B)**. **(C−D)** LGS decreases the capillary permeability of lung tissues in ALI mice. (C) Lung W/D ratio was detected by electronic scales (D) and the protein content in BALF was measured by BCA assay. **(E)** LGS decreases inflammation of lung tissues in ALI mice. The level of IL-6 in BALF was determined by ELISA. Data are represented as the mean ± SEM. ^###^ *P* < 0.001 *versus* control, *P < 0.05, **P < 0.01 and ***P < 0.001 *versus* LPS treatment by one-way ANOVA with Tukey’s test.

**Figure 7:**
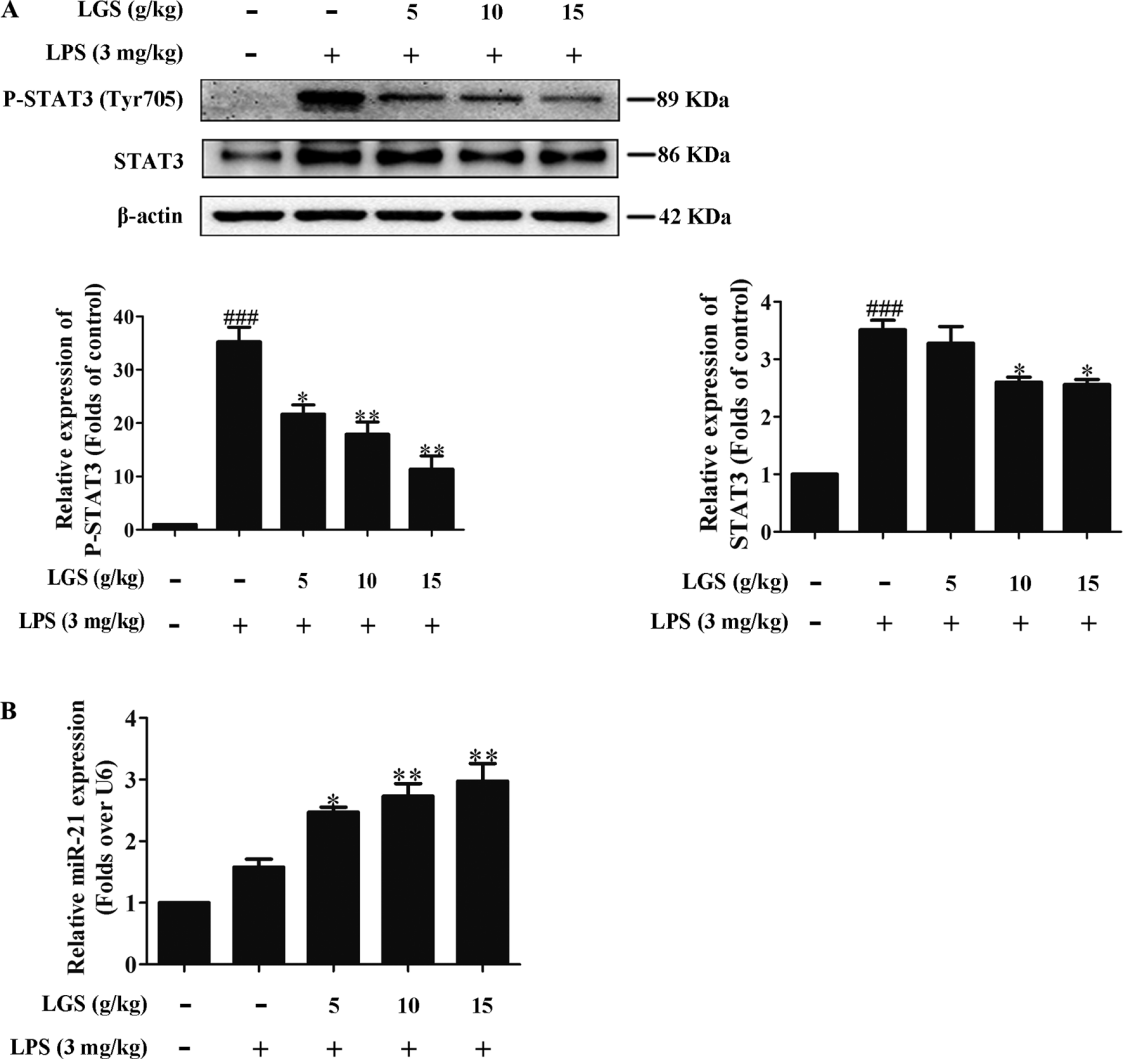
LGS inhibits the STAT3 signaling pathway by up-regulating miR-21 in ALI mice. Forty-eight mice were randomly divided into six groups each experiment. (A co-worker blinded to the experimental protocol randomized animals, n = 8/group.) After sacrifice, the lung tissues in ALI mice were harvested and the total proteins and RNA were extracted, respectively. **(A)** LGS inhibits the STAT3 signaling pathway in ALI mice. The protein expressions of STAT3 and P-STAT3 (Tyr705) were detected by Western blotting. **(B)** LGS enhances the expression of miR-21 in ALI mice. The expression of miR-21 in lung tissues was measured by qPCR. Data are represented as the mean ± SEM of three independent experiments. ^###^ *P* 0.001 *versus* control, *P < 0.05, **P < 0.01 and *versus* LPS treatment by one-way ANOVA with Tukey’s test.

We further confirmed the functional role of STAT3 and miR-21 in ALI mice. We detected that whether LGS could prevent ALI through inhibition of the STAT3 signaling pathway and up-regulation miR-21 *in vivo*. As showed in **Figures 6A, B**, lung pathologic observation showed that LGS or DEX decreased hyperemia and edema in LPS-induced ALI mice. Moreover, LGS or DEX could down-regulate the edematous indicators and inﬂammatory-associated factors, including lung W/D ratio, protein content, and IL-6 in BALF in LPS-induced ALI mice (**Figures 6C–E**). It should be noticed that LGS decrease the level of TNF-α in a dose-dependent manner *in vivo* (**Figures S5**). The release of IL-6 was more obvious than that of TNF-α after LGS treatment. We further analyzed the protein expressions of P-STAT3 (Tyr705) and STAT3 in the pulmonary tissues of ALI mice by Western blotting. As showed in **Figure 7A**, LGS suppressed the protein expressions level of STAT3 and phosphorylation of STAT3 at Tyr705. In addition, LGS significantly increased miR-21 in whole lungs (**Figure 7B**). In a summary, these results revealed that LGS inhibits activation of the STAT3 signaling pathway in LPS-induced ALI mice through up-regulating miR-21.

## Discussion

ALI remains a significant source of morbidity and mortality in the critically ill patients. Existing therapeutic strategies for ALI include decreasing release of inflammatory factors and proteins as well as suppressing organ damage induced by excessive inflammation. Despite the improvements in the understanding of the pathogenesis and treatment of this disease, the mortality of ALI caused by pneumonia or sepsis is still above 30% (Lalu et al., 2014). Thus, novel therapy strategies for ALI are necessitated.

The characteristics of ALI include excessive neutrophil migration and release of pro-inflammatory, cytotoxic mediators which are associated with inﬂammation. Accordingly, the suppression of inﬂammatory response is crucial in ALI. Clinical observation found that LGS, a famous traditional Chinese medicine formula, could inhibit ALI (Wang et al., 2011; Lee et al., 2017). In previous studies, we demonstrated that LGS significantly suppressed LPS-stimulated ALI by activating the cholinergic anti-inflammatory pathway (Liu et al., 2016), but also inhibited the protein expression of STAT3 in lung tissue of ALI rats (Liu et al., 2009).

In previous study, we noticed that the inhibition of IL-6 by LGS *in vitro* was more significant than that of TNF-α (Liu et al., 2016), which was consistent with our present research (**Figures 1B** and **S2A**). Hence, in this study, we speculated that LGS may mainly affect IL-6 related pathways, including the JAK/STAT3, the Ras/MEK/MAPK, and the PI3K/Akt pathway (Nguyen et al., 2014). In the JAK/STAT3 pathway, STAT3 is a pivotal regulator to regulate the expressions of protein genes in inﬂammation (Kim et al., 2014). The STAT3 signaling is activated by the cytokines, interferons, and growth factors binding to cell surface receptors, which leads to phosphorylation of STAT3 by JAK family phosphorylation at Tyr705 and then results in the dimerization of STAT3 from cytoplasm to nucleus to induce the transcription of corresponding gene, such as IL-6. Based on previous study (Liu et al., 2009), we further investigated whether LGS inhibits ALI through the STAT3 pathway. Our data showed that LGS could decrease STAT3, P-STAT3 (Tyr705) and suppress the dimerization and the transcriptional activity of STAT3, indicating that LGS may inhibit the STAT3 signaling pathway. Furthermore, it should be noticed that high concentrations of LGS (15 g/kg) significantly suppressed IL-6 release *in vivo* and *in vitro*, which was similar to DEX treatment. Nevertheless, DEX is an anti-inflammatory drug that has strong side effects such as alterations in body weight, atrophy of the adrenal and lymphoid organs (DaSilva et al., 2018), while LGS had no obvious toxicity in mice and patient in previous researches (Liu et al., 2016; Wang et al., 2011). MiRNAs usually bind to complementary sites in the 3-untranslated region (UTR) of target genes and regulate target gene expression by either translational inhibition, mRNA degradation, or both (Filipowicz et al., 2008). Research has proved that miRNAs are involved in inflammation. It was reported that miR-31 targeting IL-25 might regulate IL-12/23-mediated Th1/Th17 inflammatory responses during colonic inflammation process (Shi et al., 2016). MiR-15b inhibited Ring finger protein 125 and resulted in an elevation in retinoic acid-inducible gene I levels, which increased the production of pro-inflammatory cytokines and type I IFN (Zhu et al., 2015). Therefore, we further explored which miRNA regulated STAT3 mRNA in LGS treatment. Screening results showed that miR-17, miR-21, and miR-124 are highly associated with STAT3 (TargetScan, Release 7.2) and further qPCR data showed that miR-17, miR-21, and miR-124 were increased after LGS treatment. However, the up-regulation of miR-21 was most obviously. These results indicated that LGS might inhibit the STAT3 pathway through up-regulating the miR-21. Moreover, evidence has proved that the absence of miR-21 in macrophages increased the miR-21 target gene, MKK3, promoting the activation of p38-CHOP and the JNK signaling pathway, which resulted in accelerated atherosclerosis, plaque necrosis, and vascular inflammation (Canfrán-Duque et al., 2017). Studies have also demonstrated that the up-regulation of miR-21 in kidney suppressed pro-inflammatory factor programmed cell death protein 4 (PDCD4) and NF-κB activity, and increased IL-10 production in LPS-induced acute kidney inflammation (Jia et al., 2015). Recent study found that miR-21 inhibits the pro-inflammatory cytokine production in *P. gingivalis* LPS-stimulated macrophages, while miR-21 deficiency elevates the production of pro-inflammatory cytokines, suggesting the anti-inflammatory effects of miR-21 (Zhou et al., 2018). Conversely, it was reported that miR-21 up-regulated phosphorylation of AKT *via* inhibition of phosphatase and tensin homolog (PTEN), and therefore reduced the expression of ENaC-γ to aggravate inflammation in LPS-induced ALI rats (Qi et al., 2015). MiRNAs, including but not limiting miR-21, have been reported to regulate different biological functions according to the variety of cell types, organs, or diseases. Therefore, it is possible for miR-21 exerted opposite effects on mediating inflammation depending on cell types or diseases. In this study, we found that LGS up-regulated the expression level of miR-21 *in vitro* and *in vivo*. Moreover, the anti-inﬂammatory responses were attenuated by miR-21 inhibitor, demonstrating that miR-21 play a pivotal role in the anti-inﬂammatory effects of LGS. These studies demonstrated that miR-21 has a controversial role in inflammation, and required for further exploration.

Studies supported the existence of reciprocal regulation between STAT3 and miR-21. It was indicated that STAT3 is a target of miR-21 and the up-regulation of miR-21 exerts anti-inflammatory effects through suppression of the STAT signaling pathway in RAW264.7 cells (Wang et al., 2015b). On the contrary, STAT3 can activate miR-21 and miR-181b-1 *via* PTEN and CYLD, which is the switch linking inflammation to cancer in MCF10A cells (Iliopoulos et al., 2010). Likewise, studies also demonstrated that cytokine IL-21 induces activation of STAT3, and subsequently up-regulates miR-21 expression in Se’zary cells (Loffler et al., 2007; Van et al., 2011). Here, we found that the blockage of miR-21 could abolish the inhibitory effect of LGS on the STAT3 pathway, suggesting that STAT3 is a target of miR-21. It is still not clear how miR-21 down-regulates the STAT3/IL-6 signaling axis, which is required for further exploration. It is intriguing that the inhibition of P-STAT3 (Tyr705) by LGS was more obvious than that of STAT3. And we speculated that LGS attenuated phosphorylation of STAT3, but also inhibited STAT3 mRNA to block STAT3 translation, which further reduced the source of phosphorylated STAT3. It suggests that there are other modulators involved in the inhibition of p-STAT3 (Tyr705) by LGS. It has recently been reported that alpha-7 nicotinic acetylcholine receptor, BMX-ARHGAP fusion protein, and other microRNAs (including miR-204, miR-216a, etc.) could also involve in the regulation of STAT3 phosphorylation, and we speculated that these molecules may also be associated with the regulation of STAT3 by LGS, which will be further explored in our next studies (Wang et al., 2014; Wang et al., 2015a; Wazea et al., 2018).

It should be noticed that the expression of miR-21 increased after LPS stimulation *in vitro* and *in vivo*. Considered that miR-21 plays the anti-inflammatory role in our research and LPS is a well-accepted proinflammatory reagent, it seems to be controversial. However, other studies also showed that miR-21 up-regulated in primary macrophages after LPS treatment and the increase of miR-21 could not inhibit the development of inflammation (Perdiguero et al., 2011; Sheedy, 2015). Therefore, we speculated this phenomenon might be a self-emergency response to protect RAW264.7 cells from LPS-induced damage at the early stage of inflammation and the up-regulated miR-21 is insufficient to prevent the progress of inflammation.

## Conclusion

**Figure 8:**
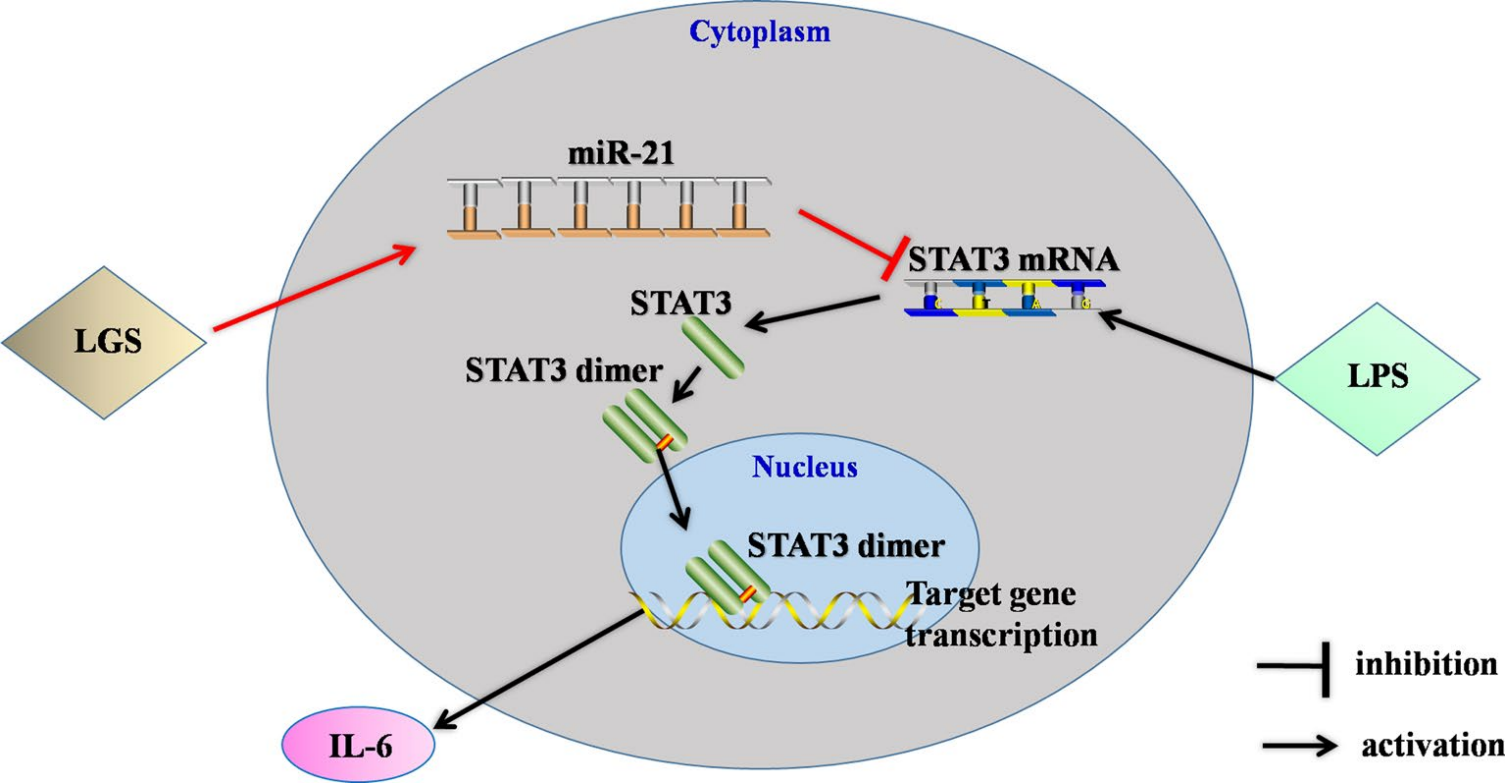
The speculated anti-inﬂammatory network of LGS. ┴ indicates an inhibitory effect. LGS exerts anti-inﬂammatory effects through up-regulation of miR-21, and subsequently inducing suppression of the STAT3 signaling pathway to decrease the release of IL-6.

In conclusion, this study showed that LGS significantly ameliorated LPS-induced ALI. We elucidated the underlying mechanism that LGS can up-regulate miR-21 to suppress STAT3 signaling and subsequently result in the decrease of IL-6 (**Figure 8**). This study provides a rationale for the clinic use of LGS in the treatment of ALI, and also reveals the critical role of miR-21 in inﬂammation which suggests that miRNAs may be an attractive therapeutic target for inflammation-mediated diseases.

## Data Availability Statement

The raw data supporting the conclusions of this manuscript will be made available by the authors, without undue reservation, to any qualified researcher.

## Ethics Statement

All experiments were executed according to the National Institutes of Health guide for the care and use of laboratory animals was approved by the ethical committee for the experimental use of animals at Southern Medical University (L2018119). Written informed consent was obtained from the owners for the participation of their animals in this study.

## Author Contributions

HY and ZL were responsible for primary data generation, analysis, and writing the manuscript. CH, YC, HC, JL, and LY participated in the design of the study. CH, YC, HC, PX, HZ, and DL were involved the *in vivo* experimentation and technical work. HC was responsible for the extensive statistical analyses. HC, and LY gave advice on the writing.

## Funding

This study was supported by the National Natural Science Foundation of China (81573671, 81730110, 81973544, 81903886), Guangdong Province Universities and Colleges Pearl River Scholar Funded Scheme (2018, JL) and Project funded by China Postdoctoral Science Foundation (2019M652988).

## Conflict of Interest

The authors declare that the research was conducted in the absence of any commercial or financial relationships that could be construed as a potential conflict of interest.

## Abbreviations

LGS, Liang-Ge-San; ALI, acute lung injury; LPS, lipopolysaccharide; ANOVA, analysis of variance; BALF, bronchoalveolar lavage fluid; BCA, bicinchoninic acid; BSA, bovine serum albumin; DAPI, 4′, 6-diamidino-2-phenylindole; DMSO, dimethyl sulfoxide; ECL, enhanced chemiluminescence; EDTA, ethylene diamine tetraacetic acid; ELISA, enzyme-linked immunosorbent assay; H & E, hematoxylin and eosin; HPLC, high-performance liquid chromatography; IL-6, interleukin-6; TNF-α, tumor necrosis factor-α; IL-1β, interleukin-1β; MTT, 3-[4,5-dimethylthiazol-2-yl]-2,5-diphenyltetrazoliumbromide; PBS, phosphate-buffered saline; qPCR, real-time quantitative polymerase chain reaction.
