# Supplementary material for: Liang-Ge-San, a Classic Traditional Chinese Medicine Formula, Attenuates Lipopolysaccharide-Induced Acute Lung Injury Through Up-Regulating miR-21
Source: Front Pharmacol. 2019 Nov 14;10:1332. doi: 10.3389/fphar.2019.01332 (PMC6868520; doi:10.3389/fphar.2019.01332)
Supplement: Supplementary file 1 [file DataSheet_1.pdf]

Supplementary Figures

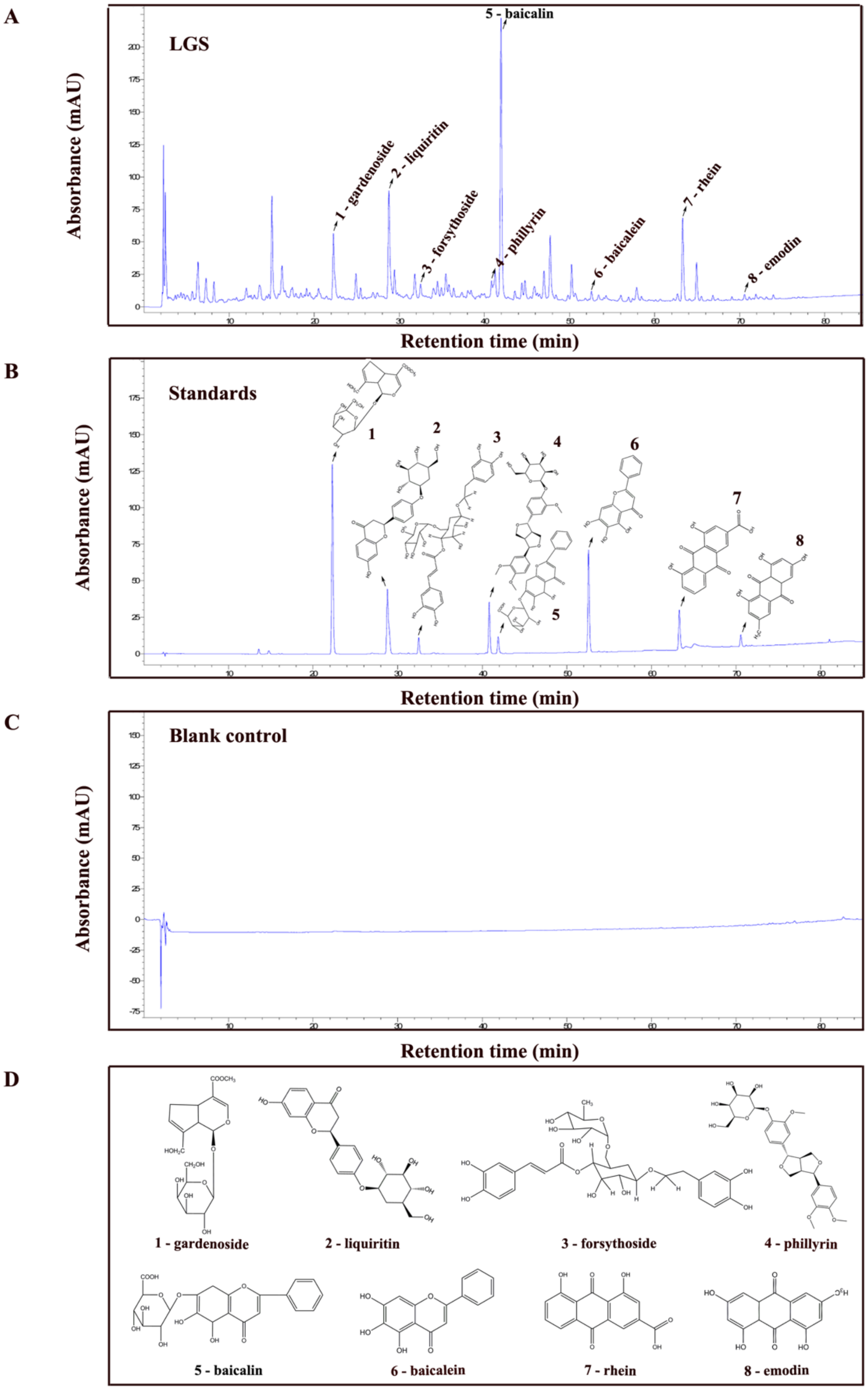

Figure S1. HPLC-DAD analysis of LGS (A) and mixed reference substances (B) as well as blank control (C) (λ=235 nm). 1 - gardenoside; 2 - liquiritin; 3 – forsythoside; 4 - phillyrin; 5 - baicalin; 6 - baicalein; 7 - rhein; 8 - emodin.

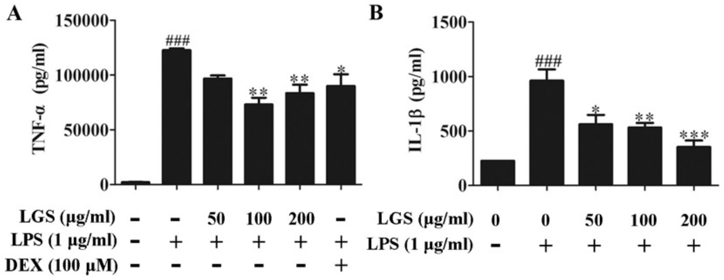

Figure S2. LGS suppresses the release of TNF-α and IL-1β in LPS-stimulated RAW 264.7 cells. (A - B) LGS inhibits the release of TNF-α and IL-1β. Macrophages were co-incubated with various concentrations of LGS (50, 100, 200 μg/ml) and LPS (1 μg/ml) for 24 h. The level of TNF-α and IL-1β in supernatants was analyzed by ELISA. Data are represented as the mean ± SEM of three independent experiments. $^{\#\#\#}P < 0.001$ *versus* control, $^{*}P < 0.05$, $^{**}P < 0.01$ and $^{***}P < 0.001$ *versus* LPS treatment by one-way ANOVA with Tukey's test.

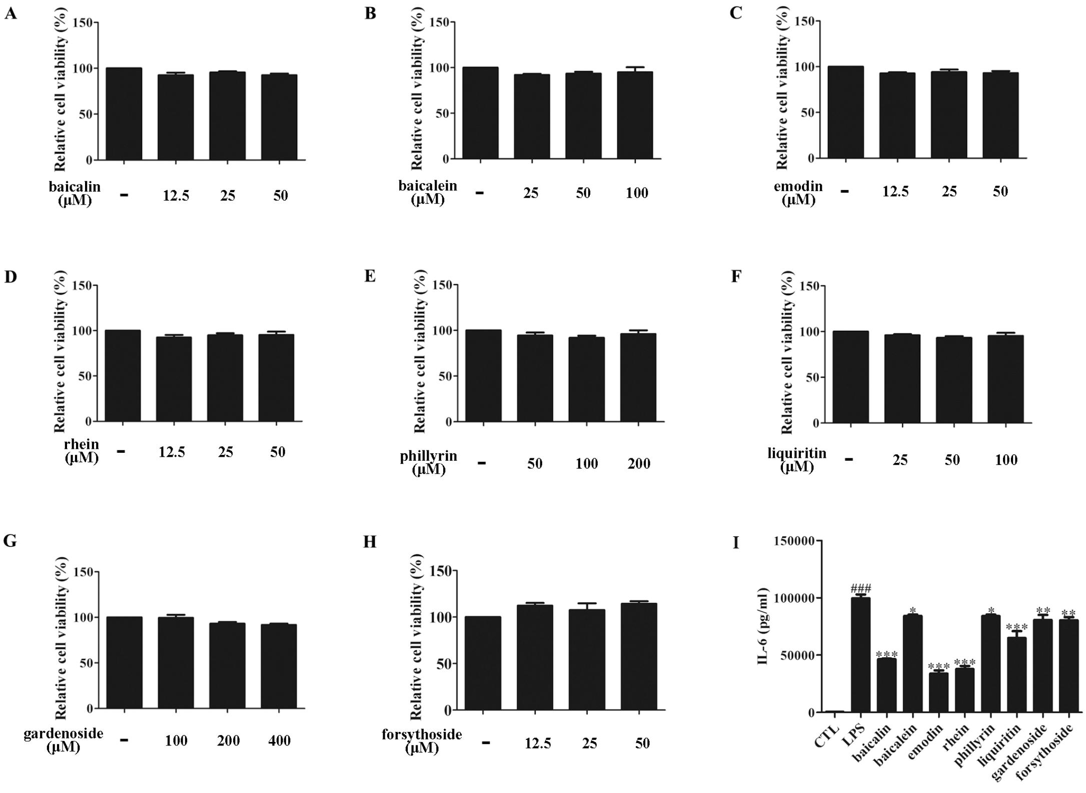

Figure S3. The anti-inflammatory activity of eight compounds in LGS. (A-G) various compounds in LGS (baicalin, baicalein, emodin, rhein, phillyrin, liquiritin and gardenoside, forsythoside, 40μM, respectively) has no obvious cytotoxicity in RAW264.7 macrophage cells. The cell cytotoxicity of LGS was confirmed by MTT assay. (H) Macrophages were co-incubated with LPS (1 μg/ml) and various compounds in LGS (baicalin, baicalein, emodin, rhein, phillyrin, liquiritin and gardenoside, forsythoside, 40μM, respectively) for 24 h. The level of IL-6 in supernatants was analyzed by ELISA. Data are represented as the mean ± SEM of three independent experiments. $^{\#\#\#}P < 0.001$ *versus* control, $^{*}P < 0.05$, $^{**}P < 0.01$ and $^{***}P < 0.001$ *versus*

LPS treatment by one-way ANOVA with Tukey's test.

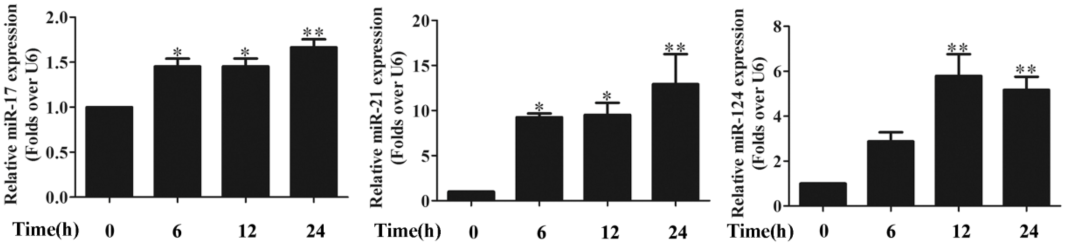

Figure S4. LGS increases the expression of miR-17, miR-21 and miR-124 in RAW264.7 cells. RAW 264.7 cells were incubated with LGS (200 μg/ml) for 6, 12 or 24 h, respectively. The expressions of miR-17, miR-21 and miR-124 were examined by qPCR. Data are represented as the mean ± SEM of three independent experiments. $^{*}P < 0.05$ and $^{**}P < 0.001$ *versus* control by one-way ANOVA with Tukey's test.

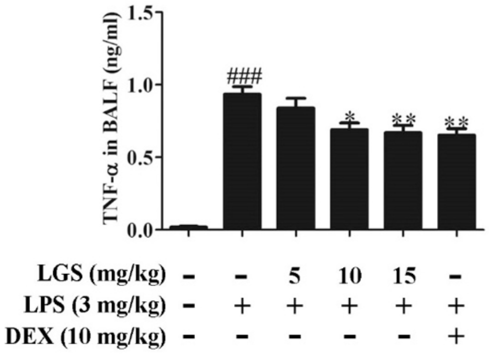

Figure S5. LGS decreases the level of TNF-α in BALF in ALI mice. The level of TNF-α was detected by ELISA. Data are represented as the mean ± SEM. $^{###}P < 0.001$ *versus* control, $^{*}P < 0.05$, $^{**}P < 0.01$ *versus* LPS treatment by one-way ANOVA with Tukey's test.

Table 1. Content of Compounds in LGS ($\bar{\chi}$±S, n=3)

Compounds	baicalin	baicalein	emodin	rhein	phillyrin	liquiritin	gardenoside	frosythoside
Concentration (μg/10 μL)	2.518±0.162	0.0608±0.05	0.0655±0.04	0.665±0.07	0.279±0.006	0.896±0.111	0.5068±0.008	0.227±0.005

*Data were presented as mean ± S.E. The experiments were performed at least three times.

康美药业股份有限公司
中药饮片检验报告

报告书编号：BM201712010010

检品名称	大黄	产　　地	四川
检品来源	车间	包装规格	统装
数　　量	/	批　　号	171103591
取 样 量	200g	请 检 人	丁望星
检验目的	入库检验	生产日期	/
请检日期	2017 年 11 月 23 日	报告日期	2017 年 12 月 01 日
检验依据	《中国药典》2015 年版一部		

检验项目	标准规定	检验结果	结论
【性状】	应具有大黄的性状特征	具有大黄的性状特征	符合规定
【鉴别】			
显微鉴别*	应具有大黄的显微特征	具有大黄的显微特征	符合规定
理化鉴别*	应呈正反应	呈正反应	符合规定
薄层鉴别*	供试品色谱中，在与对照药材色谱相应的位置上，应显相同的五个橙黄色荧光主斑点；在与对照品色谱相应的位置上，应显相同的橙黄色荧光斑点，置氨蒸气中熏后，斑点应变为红色	供试品色谱中，在与对照药材色谱相应的位置上，显相同的五个橙黄色荧光主斑点；在与对照品色谱相应的位置上，显相同的橙黄色荧光斑点，置氨蒸气中熏后，斑点变为红色	符合规定
【检查】			
药屑、杂质	不得过 3%	0.2%	符合规定
土大黄苷*	供试品色谱中，在与对照品色谱相应的位置上，应不得显相同的亮蓝色荧光斑点	供试品色谱中，在与对照品色谱相应的位置上，不得显相同的亮蓝色荧光斑点	符合规定
干燥失重	不得过 15.0%	7.4%	符合规定
总灰分*	不得过 10.0%	8.1%	符合规定
二氧化硫残留量*	不得过 150mg/kg	0mg/kg	符合规定
【浸出物】	不得少于 25.0%	38.2%	符合规定
【含量测定】			
总蒽醌	本品按干燥品计算，含总蒽醌以芦荟大黄素($C_{15}H_{10}O_5$)、大黄酸($C_{15}H_8O_6$)、大黄素($C_{15}H_{10}O_5$)、大黄酚($C_{15}H_{10}O_4$)和大黄素甲醚($C_{16}H_{12}O_5$)的总量计，不得少于 1.5%	2.7%	符合规定
检验结论	本品按《中国药典》2015 年版一部检验，结果符合规定。		
备　　注	带“*”的检测项目为引用中药材检验数据；检验报告书未加盖检验报告专用章无效		

检验人：周匡　　复核人：[illegible]　　质量控制负责人：[illegible]

康美药业股份有限公司
中药饮片检验报告

报告书编号：BM201712010010

检品名称	大黄	产　　地	四川
检品来源	车间	包装规格	统装
数　　量	/	批　　号	171103591
取 样 量	200g	请 检 人	丁望星
检验目的	入库检验	生产日期	/
请检日期	2017 年 11 月 23 日	报告日期	2017 年 12 月 01 日
检验依据	《中国药典》2015 年版一部		

检验项目	标准规定	检验结果	结论
游离蒽醌	本品按干燥品计算，含游离蒽醌以芦荟大黄素（$C_{15}H_{10}O_5$）、大黄酸（$C_{15}H_8O_6$）、大黄素（$C_{15}H_{10}O_5$）、大黄酚（$C_{15}H_{10}O_4$）和大黄素甲醚（$C_{16}H_{12}O_5$）的总量计，不得少于 0.35%	1.7%	符合规定

以下空白

检验结论	本品按《中国药典》2015 年版一部检验，结果符合规定。
备　　注	带“*”的检测项目为引用中药材检验数据；检验报告书未加盖检验报告专用章无效

检验人：周佳　　复核人：[illegible]　　质量控制负责人：[illegible]

康美药业股份有限公司
中药饮片检验报告

报告书编号：BM201711160069

检品名称	连翘（青翘）	产　地	山西
检品来源	车间	包装规格	统装
数　量	/	批　号	171102131
取样量	200g	请检人	丁望星
检验目的	入库检验	生产日期	/
请检日期	2017 年 11 月 16 日	报告日期	2017 年 11 月 16 日
检验依据	《中国药典》2015 年版一部		

检验项目	标准规定	检验结果	结论
【性状】*	应具有连翘的性状特征	具有连翘的性状特征	符合规定
【鉴别】			
显微鉴别*	应具有连翘的显微特征	具有连翘的显微特征	符合规定
薄层鉴别*	供试品色谱中，在与对照药材色谱和对照品色谱相应的位置上，应显相同颜色的斑点	供试品色谱中，在与对照药材色谱和对照品色谱相应的位置上，显相同颜色的斑点	符合规定
【检查】			
药屑、杂质	不得过 3%	0.2%	符合规定
水分	不得过 10.0%	8.3%	符合规定
总灰分*	不得过 4.0%	3.0%	符合规定
二氧化硫残留量*	不得过 150mg/kg	0mg/kg	符合规定
【浸出物】*	不得少于 30.0%	41.2%	符合规定
【含量测定】*	本品按干燥品计算，含连翘苷($C_{27}H_{34}O_{17}$)不得少于 0.15%	0.70%	符合规定
	本品按干燥品计算，含连翘酯苷 A($C_{29}H_{36}O_{15}$)不得少于 0.25%	16%	符合规定

以下空白

检验结论	本品按《中国药典》2015 年版一部检验，结果符合规定。
备　注	带"*"的检测项目为引用中药材检验数据；检验报告书未加盖检验报告专用章无效

检验人：吴妹泥　　复核人：林颖　　质量控制负责人：[illegible]

康美药业股份有限公司

中药饮片检验报告

报告书编号：BM2018022300014

康美药业股份有限公司（2）检验报告专用章

检品名称	黄芩片	产　　地	河北
检品来源	车间	包装规格	统装
数　　量	/	批　　号	180106211
取 样 量	200g	请 检 人	黄勇泉
检验目的	入库检验	生产日期	/
请检日期	2018 年 02 月 01 日	报告日期	2018 年 02 月 23 日
检验依据	《中国药典》2015 年版一部及《国家食品药品监督管理局药品检验补充检验方法和检验项目批准件》2010004 号		

检验项目	标准规定	检验结果	结论
【性状】	应具有黄芩片的性状特征	具有黄芩片的性状特征	符合规定
【鉴别】			
显微鉴别*	应具有黄芩的显微特征	具有黄芩的显微特征	符合规定
薄层鉴别*	供试品色谱中，在与对照药材色谱相应的位置上，应显相同颜色的斑点；在与对照品色谱相应的位置上，应显三个相同的暗色斑点	供试品色谱中，在与对照药材色谱相应的位置上，显相同颜色的斑点；在与对照品色谱相应的位置上，显三个相同的暗色斑点	符合规定
【检查】			
药屑、杂质	不得过 3%	0.2%	符合规定
水分	不得过 13%	6%	符合规定
二氧化硫残留量*	不得过 150mg/kg	0mg/kg	符合规定
金胺 O*	不得检出	未检出	符合规定
【含量测定】	本品按干燥品计算，含黄芩苷（$C_{21}H_{18}O_{11}$）不得少于 8.0%	21%	符合规定

以下空白

检验结论	本品按《中国药典》2015 年版一部及《国家食品药品监督管理局药品检验补充检验方法和检验项目批准件》2010004 号检验，结果符合规定。
备　　注	带“*”的检测项目为引用中药材检验数据；检验报告书未加盖检验报告专用章无效

检验人：周连　　复核人：林毅　　质量控制负责人：[illegible]

康美药业股份有限公司
中药饮片检验报告

报告书编号：BM201712260033

检品名称	甘草片	产　　地	内蒙古
检品来源	车间	包装规格	统装
数　　量	/	批　　号	171201291
取 样 量	200g	请 检 人	张炜全
检验目的	入库检验	生产日期	/
请检日期	2017 年 12 月 12 日	报告日期	2017 年 12 月 26 日
检验依据	《中国药典》2015 年版一部		

检验项目	标准规定	检验结果	结论
【性状】	应具有甘草片的性状特征	具有甘草片的性状特征	符合规定
【鉴别】			
显微鉴别*	应具有甘草的显微特征	具有甘草的显微特征	符合规定
薄层鉴别*	供试品色谱中，在与对照药材色谱相应的位置上，应显相同颜色的荧光斑点；在与对照品色谱相应的位置上，应显相同的橙黄色荧光斑点	供试品色谱中，在与对照药材色谱相应的位置上，显相同颜色的荧光斑点；在与对照品色谱相应的位置上，显相同的橙黄色荧光斑点	符合规定
【检查】			
药屑、杂质	不得过 3%	0.2%	符合规定
水分	不得过 12.0%	9.6%	符合规定
总灰分*	不得过 5.0%	3.7%	符合规定
重金属及有害元素*	铅不得过 5mg/kg	0.1mg/kg	符合规定
	镉不得过 0.3mg/kg	0.02mg/kg	符合规定
	砷不得过 2mg/kg	0.1mg/kg	符合规定
	汞不得过 0.2mg/kg	未检出	符合规定
	铜不得过 20mg/kg	6.0mg/kg	符合规定
二氧化硫残留量*	不得过 150mg/kg	0mg/kg	符合规定
【含量测定】	本品按干燥品计算，含甘草苷($C_{21}H_{22}O_9$)不得少于 0.45%	0.72%	符合规定
	本品按干燥品计算，含甘草酸($C_{42}H_{62}O_{16}$)不得少于 1.8%	2.2%	符合规定
以下空白			
检验结论	本品按《中国药典》2015 年版一部检验，结果符合规定。		
备　　注	带“*”的检测项目为引用中药材检验数据；检验报告书未加盖检验报告专用章无效		

检验人：钟炯哲　　复核人：林毅　　质量控制负责人：[illegible]

康美药业股份有限公司
中药饮片检验报告

报告书编号：BM201712100015

检品名称	栀子	产　　地	江西
检品来源	车间	包装规格	统装
数　　量	/	批　　号	171107691
取 样 量	200g	请 检 人	张炜全
检验目的	入库检验	生产日期	/
请检日期	2017 年 12 月 10 日	报告日期	2017 年 12 月 10 日
检验依据	《中国药典》2015 年版一部		

检验项目	标准规定	检验结果	结论
【性状】	应具有栀子的性状特征	具有栀子的性状特征	符合规定
【鉴别】			
显微鉴别*	应具有栀子的显微特征	具有栀子的显微特征	符合规定
薄层鉴别*	供试品色谱中，在于对照药材色谱和对照品色谱相应的位置上，应显相同颜色的斑点	供试品色谱中，在于对照药材色谱和对照品色谱相应的位置上，显相同颜色的斑点	符合规定
【检查】			
药屑、杂质	不得过 3%	0.3%	符合规定
水分	不得过 8.5%	6.2%	符合规定
总灰分*	不得过 6.0%	4.1%	符合规定
二氧化硫残留量*	不得过 150mg/kg	0mg/kg	符合规定
【含量测定】*	本品按干燥品计算，含栀子苷（$C_{17}H_{24}O_{10}$）不得少于 1.8%	6.3%	符合规定

以下空白

检验结论	本品按《中国药典》2015 年版一部检验，结果符合规定。
备　　注	带“*”的检测项目为引用中药材检验数据；检验报告书未加盖检验报告专用章无效

检验人：[signature]　　复核人：[signature]　　质量控制负责人：[signature]

（印章：康美药业股份有限公司（2）检验报告专用章）

康美药业股份有限公司
中药饮片检验报告

报告书编号：BM201712280015

检品名称	芒硝	产　　地	四川
检品来源	车间	包装规格	统装
数　　量	/	批　　号	171207391
取 样 量	200g	请 检 人	丁望星
检验目的	入库检验	生产日期	/
请检日期	2017 年 12 月 28 日	报告日期	2017 年 12 月 28 日
检验依据	《中国药典》2015 年版一部		

检验项目	标准规定	检验结果	结论
【性状】*	应具有芒硝的性状特征	具有芒硝的性状特征	符合规定
【鉴别】			
理化鉴别*	应呈正反应	呈正反应	符合规定
	应呈正反应	呈正反应	符合规定
【检查】			
药屑、杂质	不得过 3%	0.1%	符合规定
干燥失重*	应为 51.0%～57.0%	55.5%	符合规定
铁盐与锌盐*	不得发生浑浊或显蓝色	符合规定	符合规定
镁盐*	不得发生浑浊	符合规定	符合规定
重金属*	不得过 10mg/kg	符合规定	符合规定
砷盐*	不得过 10mg/kg	符合规定	符合规定
【含量测定】*	本品按干燥品计算，含硫酸钠（Na_2SO_4）不得少于 99.0%	99.2%	符合规定

以下空白

检验结论	本品按《中国药典》2015 年版一部检验，结果符合规定。
备　　注	带“*”的检测项目为引用中药材检验数据；检验报告书未加盖检验报告专用章无效

检验人：[signature]　　复核人：[signature]　　质量控制负责人：[signature]

康美药业股份有限公司
中药饮片检验报告

报告书编号：BM201801150008

检品名称	薄荷	产　地	江苏
检品来源	车间	包装规格	统装
数　量	/	批　号	171207731
取 样 量	200g	请 检 人	黄勇泉
检验目的	入库检验	生产日期	/
请检日期	2018 年 01 月 09 日	报告日期	2018 年 01 月 15 日
检验依据	《中国药典》2015 年版一部		

检验项目	标准规定	检验结果	结论
【性状】	应具有薄荷的性状特征	具有薄荷的性状特征	符合规定
【鉴别】			
显微鉴别*	应具有薄荷的显微特征	具有薄荷的显微特征	符合规定
理化鉴别*	应呈正反应	呈正反应	符合规定
薄层鉴别*	供试品色谱中，在与对照药材色谱和对照品色谱相应的位置上，应显相同颜色的斑点	供试品色谱中，在与对照药材色谱和对照品色谱相应的位置上，显相同颜色的斑点	符合规定
【检查】			
药屑、杂质	不得过 3%	0.3%	符合规定
水分	不得过 13.0%	8.1%	符合规定
总灰分*	不得过 11.0%	9.3%	符合规定
酸不溶性灰分*	不得过 3.0%	2.2%	符合规定
二氧化硫残留量*	不得过 150mg/kg	0mg/kg	符合规定
【含量测定】	本品含挥发油不得少于 0.40%（ml/g）	2.2%（ml/g）	符合规定

以下空白

检验结论	本品按《中国药典》2015 年版一部检验，结果符合规定。
备　注	带“*”的检测项目为引用中药材检验数据；检验报告书未加盖检验报告专用章无效

检验人：[signature]　　复核人：[signature]　　质量控制负责人：[signature]
